# Supplementary material for: SALL4 Is Required for YAP1-Dependent Malignant and Regenerative Hepatocyte-to-Cholangiocyte Reprogramming
Source: Cancer Res Commun. 2025 Sep 25;5(9):1714–27. doi: 10.1158/2767-9764.CRC-25-0172 (PMC12462609; doi:10.1158/2767-9764.CRC-25-0172)
Supplement: Supplementary Figure S1 [file crc-25-0172_supplementary_figure_s1_suppsf1.docx]

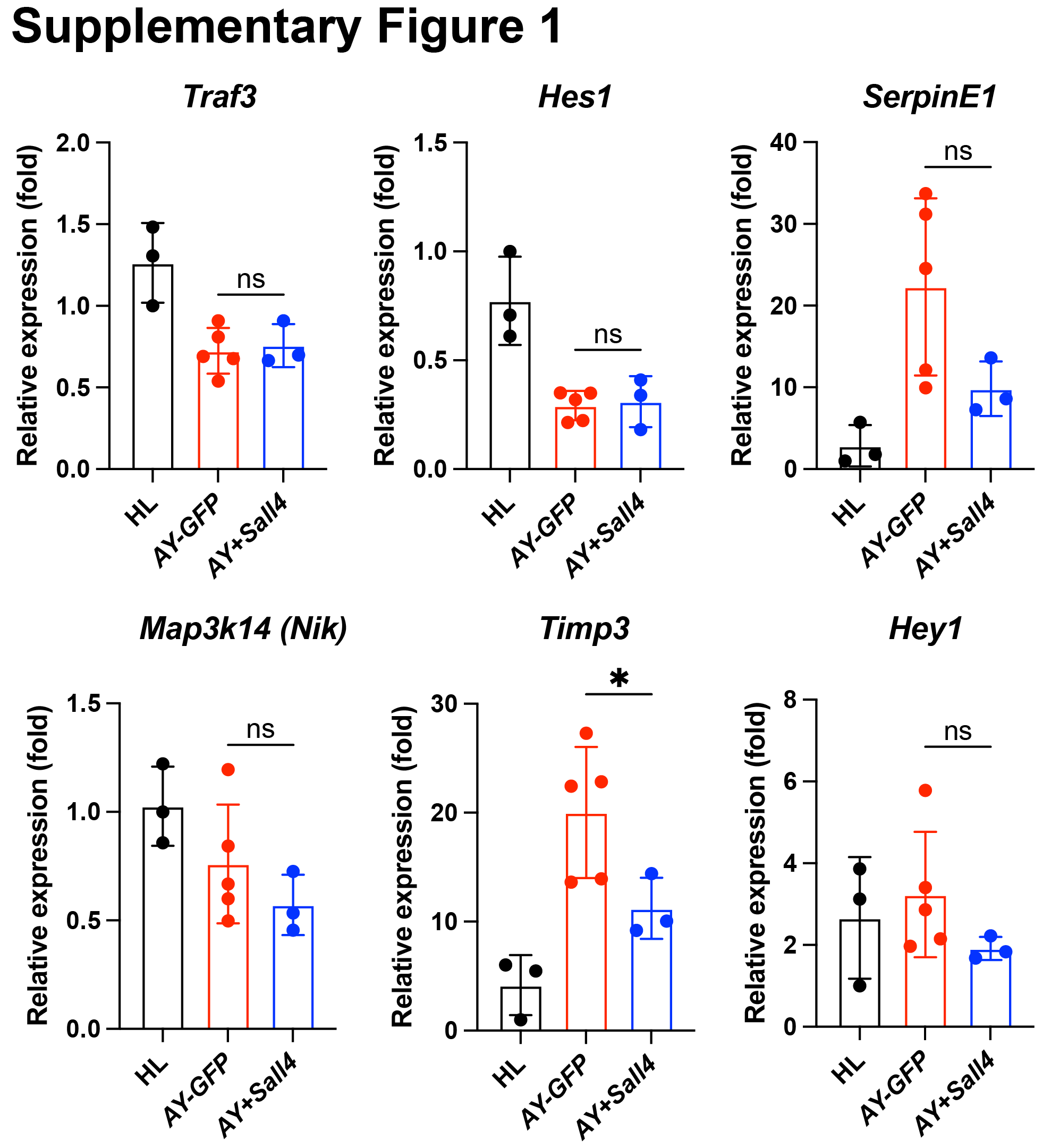


**Supplementary Figure 1. SALL4 overexpression suppresses *Timp3* expression but has limited impact on Notch- and TRAF3/NIK–related genes in AY-driven CCA.**

qRT-PCR analysis for indicated genes related to the Notch (*Hes1, Hey1*), TRAF3/NIK (*Traf3, Map3k14*) and TGF-β (*SerpinE1, Timp3*) pathways in liver tissues from healthy liver (HL), *AY-GFP*, and *AY-Sall4* groups at 2.5 wk post-HDTVI. Relative mRNA expression levels were normalized to housekeeping genes (*Gapdh*) and presented as fold changes relative to HL. SALL4 overexpression significantly reduced *Timp3* expression compared to the AY-GFP group (*p < 0.05). No significant differences (ns) were observed in the expression of the other genes. Data are shown as mean ± CD (n = 3–5 per group). Statistical significance was determined by Student’s t-test.
