# Supplementary material for: SALL4 Is Required for YAP1-Dependent Malignant and Regenerative Hepatocyte-to-Cholangiocyte Reprogramming
Source: Cancer Res Commun. 2025 Sep 25;5(9):1714–27. doi: 10.1158/2767-9764.CRC-25-0172 (PMC12462609; doi:10.1158/2767-9764.CRC-25-0172)
Supplement: Supplementary Table S1 — Lists and the quantity of plasmids for HDTVI [file crc-25-0172_supplementary_table_s1_suppst1.docx]

**Supplementary table 1. Lists and the quantity of plasmids for HDTVI**

| **Model** | **Plasmids** | **µg / 2 mL** | **Source** | **RRID** |
| --- | --- | --- | --- | --- |
| *AY*  (CCA) | *pT3-EF1α-myrAkt-HA* | 10 | Addgene# 179909 | RRID:Addgene_179909 |
|  | *pT3-EF1α-YAP1^S127A^* | 20 | Addgene# 86497 | RRID:Addgene_86497 |
|  | *pCMV-SB* | 1.2 | Previous study^1^ |  |
| *ANRAS*  (cHCC-CCA) | *pT3-EF1α-myrAkt-HA* | 10 | Addgene# 179909 | RRID:Addgene_179909 |
|  | *pT3-EF1α-NRAS^G12V^* | 20 | *Previous study^2^* |  |
|  | *pCMV-SB* | 1.2 | *Previous study^1^* |  |
| *KP53*  (mHCC/CCA) | *pT3-EF1α-KRAS^G12D^-HA* | 5 | *Previous study^3^* |  |
|  | *pT3- EF1α -shp53-GFP* | 10 | *Previous study^3^* |  |
|  | *pCMV-SB* | 0.6 | *Previous study^1^* |  |
| *AY + pT3-Sall4* | *pT3-EF1α-myrAkt-HA* | 10 | Addgene# 179909 | RRID:Addgene_179909 |
|  | *pT3-EF1α-YAP1^S127A^* | 20 | Addgene# 86497 | RRID:Addgene_86497 |
|  | *pT3-EF1α-Sall4-V5 / pT3-EF1α-GFP* | 20 | This study |  |
|  | *pCMV-SB* | 2 | *Previous study^1^* |  |
| *AY + pT3-BMI1* | *pT3-EF1α-myrAkt-HA* | 10 | Addgene# 179909 | RRID:Addgene_179909 |
|  | *pT3-EF1α-YAP1^S127A^* | 20 | Addgene# 86497 | RRID:Addgene_86497 |
|  | *pT3-EF1α-BMI1-V5 / pT3-EF1α-GFP* | 20 | This study |  |
|  | *pCMV-SB* | 2 | *Previous study^1^* |  |
| *AY + sgBmi1* | *pT3-EF1α-myrAkt-HA* | 10 | Addgene# 179909 | RRID:Addgene_179909 |
|  | *pT3-EF1α-YAP1^S127A^* | 20 | Addgene# 86497 | RRID:Addgene_86497 |
|  | *SB-LSL-Cas9-sgBmi1 / sgEmpty* | 20 | This study |  |
|  | *Cre-P2A-tdTomato* | 20 | Addgene# 176837 | RRID:Addgene_176837 |
|  | *pCMV-SB* | 2.8 | *Previous study^1^* |  |

[1] Wang J, Dong M, Xu Z, Song X, Zhang S, Qiao Y, Che L, Gordan J, Hu K, Liu Y, Calvisi DF, Chen X: Notch2 controls hepatocyte-derived cholangiocarcinoma formation in mice. Oncogene 2018, 37:3229-42.

[2] Ho C, Wang C, Mattu S, Destefanis G, Ladu S, Delogu S, Armbruster J, Fan L, Lee SA, Jiang L, Dombrowski F, Evert M, Chen X, Calvisi DF: AKT (v-akt murine thymoma viral oncogene homolog 1) and N-Ras (neuroblastoma ras viral oncogene homolog) coactivation in the mouse liver promotes rapid carcinogenesis by way of mTOR (mammalian target of rapamycin complex 1), FOXM1 (forkhead box M1)/SKP2, and c-Myc pathways. Hepatology 2012, 55:833-45.

[3] Hu S, Molina L, Tao J, Liu S, Hassan M, Singh S, Poddar M, Bell A, Sia D, Oertel M, Raeman R, Nejak-Bowen K, Singhi A, Luo J, Monga SP, Ko S: NOTCH-YAP1/TEAD-DNMT1 Axis Drives Hepatocyte Reprogramming Into Intrahepatic Cholangiocarcinoma. Gastroenterology 2022, 163:449-65.
