# Supplementary material for: SALL4 Is Required for YAP1-Dependent Malignant and Regenerative Hepatocyte-to-Cholangiocyte Reprogramming
Source: Cancer Res Commun. 2025 Sep 25;5(9):1714–27. doi: 10.1158/2767-9764.CRC-25-0172 (PMC12462609; doi:10.1158/2767-9764.CRC-25-0172)
Supplement: Supplementary Table S2 — Primary & Secondary antibodies [file crc-25-0172_supplementary_table_s2_suppst2.docx]

**Supplementary table 2. Primary & Secondary antibodies**

| **Name** | **Manufacturer** | **Cat#** | **RRID** | **Source** | **Dilution** |
| --- | --- | --- | --- | --- | --- |
| **Primary antibodies** | | | | | |
| HA-tag | Cell Signaling Technology | 2367S | RRID:AB_10691311 | Mouse | 1:200 |
| HNF4A | Perseus Proteomics | PP-H1415-0C | RRID:AB_3659607 | Mouse | 1:200 |
| Ki67 | Cell Signaling Technology | 12202S | RRID:AB_2620142 | Rabbit | 1:500 |
| panCK | Agilent | Z0622 | RRID:AB_2650434 | Rabbit | 1:200 |
| V5-tag | eBioscience | 12-6796-82 | RRID:AB_10718239 | Mouse | 1:100 |
| SOX9 | Sigma-Aldrich | AB5535 | RRID:AB_2239761 | Rabbit | 1:2000 |
| CK19 | DHSB | TROMA-III-s | RRID:AB_2133570 | Rat | 1:10 |
| GFP | Cell Signaling Technology | 2956S | RRID:AB_1196615 | Rabbit | 1:200 |
| CD45 | Cell Signaling Technology | 55307S |  | Rat | 1:100 |
| ɑ-SMA | Abcam | ab-5694 | RRID:AB_2223021 | Rabbit | 1:500 |
| **Secondary antibodies** | | | | | |
| Donkey anti-Rabbit IgG Biotin | Sigma-Aldrich | AP182B | RRID:AB_92587 | Donkey | 1:250 |
| Goat anti-Mouse IgG  Biotin | Sigma-Aldrich | AP181B | RRID:AB_92577 | Goat | 1:250 |
| Goat anti-Rat IgG Biotin | Sigma-Aldrich | AP183B | RRID:AB_92595 | Goat | 1:250 |
| Alexa Fluor 488 Donkey Anti-Rat IgG | Jackson Immuno Research Labs | 712-545-153 | RRID:AB_2340684 | Donkey | 1:500 |
| Alexa Fluor 555 Donkey Anti-Rabbit IgG | Invitrogen | A31572 | RRID:AB_162543 | Donkey | 1:500 |
| Alexa Fluor 647 Donkey Anti-Mouse IgG | Jackson Immuno Research Labs | 715-605-151 | RRID:AB_2340863 | Donkey | 1:500 |
| Hoechst 33258 | Sigma-Aldrich | B-2883 |  |  |  |
