# Supplementary material for: SALL4 Is Required for YAP1-Dependent Malignant and Regenerative Hepatocyte-to-Cholangiocyte Reprogramming
Source: Cancer Res Commun. 2025 Sep 25;5(9):1714–27. doi: 10.1158/2767-9764.CRC-25-0172 (PMC12462609; doi:10.1158/2767-9764.CRC-25-0172)
Supplement: Supplementary Table S3 — Primers and plasmids for the cloning Plasmids [file crc-25-0172_supplementary_table_s3_suppst3.docx]

**Supplementary table 3. Primers and plasmids for the cloning**

| **Plasmids** | | |
| --- | --- | --- |
| **Name** | **Source** | |
| *pT3-EH1αH-myrAkt* | Addgene, 179909, RRID:Addgene_179909 | |
| *pT3-EH1α-BMI1-V5* | Addgene, 31783, RRID:Addgene_31783 | |
| *pENTR223.1-Sall4* | DNASU, MmCD00083348 | |
| *SB-LSL-Cas9-sgRNA* | *Previous study^1^* | |
| **Primers** | | |
| **Sequences** | | **Name** |
| AGTCGACTGGATCCGGTACCGCCACCATGCATCGAACAACGAGAATCAAGATCACT | | HiFi-BMI1-F |
| AGTTTGGACAAACCACAACTAGAATGCAGCttaACCAGAAGAAGTTGCTGATGACCCAT | | HiFi-BMI1-R |
| GTCGACTGGATCCGGTACCGCCACCATGTCGAGGCGCAAGCAGGCGAAGCCCCA | | HiFi-Sall4-F |
| AGTTTGGACAAACCACAACTAGAATGCAGCttaCGTAGAATCGAGACCGAGGAGAGGGTTAGGGATAGGCTTACCGCTGACAGCAATCTTATTTTCCTCCAG | | HiFi-Sall4-R |
| CACCGATTGACGTCATGTATGAAG | | sgBmi1#1-F |
| AAACCTTCATACATGACGTCAATC | | sgBmi1#1-R |
| CACCGGCCGAACTCTGTATTTCAA | | sgBmi1#2-F |
| AAACTTGAAATACAGAGTTCGGCC | | sgBmi1#2-R |

**Supplementary table 4. Primers for qRT-PCR**

| **Gene** |  | **Sequences** |
| --- | --- | --- |
| *Bmi1* | F | TGCTGGAGAGCTGGAAAGTG |
|  | R | GGACTGGGCAAACAGGAAGA |
| *Sall4* | F | ATTCGCGTCCAGGTGAACAT |
|  | R | GACACGGACACTTGCTGAGA |
| *Hprt* | F | CAGTCCCAGCGTCGTGATTA |
|  | R | CACTTTTTCCAAATCCTCGGCA |
| *Gapdh* | F | TGTGAACGGATTTGGCCGTA |
|  | R | ATGAAGGGGTCGTTGATGGC |
| *Hes1* | F | ATGACTGTGAAGCACCTCCG |
|  | R | CGTTCATGCACTCGCTGAAG |
| *Hey1* | F | GCCTGGTCTCCCATCTCAAC |
|  | R | TGTGTGGGTGATGTCCGAAG |
| *Map3k14* | F | GAGTTCAGCCCCACCTTCTC |
|  | R | GTTGCATGGGCCACATTGTT |
| *Serpine1* | F | CCGATGGGCTCGAGTATGAC |
|  | R | TCCAAGATGTTGGTGAGGGC |
| *Timp3* | F | CCAGAACCGCAGTGAAGAGT |
|  | R | GTACGCCAGGGAACCAAGAA |
| *Traf3* | F | CGTGCCGACTGCAAAGAAAA |
|  | R | TTGATCATGGGCACTTGGCT |

[1] Park Y, Hu S, Kim M, Oertel M, Singhi A, Monga SP, Liu S, Ko S: Context-Dependent Distinct Roles of SOX9 in Combined Hepatocellular Carcinoma-Cholangiocarcinoma. Cells 2024, 13.
