# Supplementary material for: SALL4 Is Required for YAP1-Dependent Malignant and Regenerative Hepatocyte-to-Cholangiocyte Reprogramming
Source: Cancer Res Commun. 2025 Sep 25;5(9):1714–27. doi: 10.1158/2767-9764.CRC-25-0172 (PMC12462609; doi:10.1158/2767-9764.CRC-25-0172)
Supplement: Supplementary Table S4 — Primers for qRT-PCR [file crc-25-0172_supplementary_table_s4_suppst4.docx]

**Supplementary table 4. Primers for qRT-PCR**

| **Gene** |  | **Sequences** |
| --- | --- | --- |
| *Bmi1* | F | TGCTGGAGAGCTGGAAAGTG |
|  | R | GGACTGGGCAAACAGGAAGA |
| *Sall4* | F | ATTCGCGTCCAGGTGAACAT |
|  | R | GACACGGACACTTGCTGAGA |
| *Hprt* | F | CAGTCCCAGCGTCGTGATTA |
|  | R | CACTTTTTCCAAATCCTCGGCA |
| *Gapdh* | F | TGTGAACGGATTTGGCCGTA |
|  | R | ATGAAGGGGTCGTTGATGGC |
| *Hes1* | F | ATGACTGTGAAGCACCTCCG |
|  | R | CGTTCATGCACTCGCTGAAG |
| *Hey1* | F | GCCTGGTCTCCCATCTCAAC |
|  | R | TGTGTGGGTGATGTCCGAAG |
| *Map3k14* | F | GAGTTCAGCCCCACCTTCTC |
|  | R | GTTGCATGGGCCACATTGTT |
| *Serpine1* | F | CCGATGGGCTCGAGTATGAC |
|  | R | TCCAAGATGTTGGTGAGGGC |
| *Timp3* | F | CCAGAACCGCAGTGAAGAGT |
|  | R | GTACGCCAGGGAACCAAGAA |
| *Traf3* | F | CGTGCCGACTGCAAAGAAAA |
|  | R | TTGATCATGGGCACTTGGCT |
